# Optic Neuritis: From Magnocellular to Cognitive Residual Dysfunction

**DOI:** 10.3233/BEN-120322

**Published:** 2013

**Authors:** Anne-Claire Viret, Céline Cavézian, Olivier Coubard, Vivien Vasseur, Noa Raz, Netta Levin, Catherine Vignal, Olivier Gout, Sylvie Chokron

**Affiliations:** ^1^Unité Fonctionnelle Vision et Cognition, Fondation Ophtalmologique Rothschild, ParisFrance; ^2^Service de Neurologie, Fondation Ophtalmologique Rothschild, ParisFrance; ^3^Laboratoire Vision, Action, Cognition—EAU 01, Université Paris Descartes—Sorbonne Paris Cité, Boulogne-Billancourt, FranceFrance; ^4^Service d’Ophtalmologie, Fondation Ophtalmologique Rothschild, ParisFrance; ^5^The Neuropsychological Laboratory, CNS-Fed, ParisFrance; ^6^Department of Neurology, Hadassah Hebrew-University Hospital, JerusalemIsrael; ^7^Laboratoire de Psychologie de la Perception, UMR 8158, CNRS and Université Paris-Descartes, ParisFrance

**Keywords:** Optic neuritis, spatial frequency, magnocellular pathway, visual cognition

## Abstract

Optic Neuritis (ON) has been associated to both parvocellular dysfunction and to an alteration of the magnocellular pathway. After objective visual field and acuity recovery, ON patients may complain about their vision suggesting a residual subclinical deficit. To better characterize visual abnormalities, 8 patients recovering from a first ON episode as well as 16 healthy controls performed a simple detection task and a more complex categorization task of images presented in low spatial frequencies (to target the magnocellular system) or in high spatial frequencies (to target the parvocellular system) or of non-filtered images. When completing the tasks with their (previously) pathologic eye, optic neuritis patients showed lower accuracy compared to controls or to their healthy eye for low spatial frequency images only. Conjointly, the longest reaction times were observed with the previously pathologic eye regardless the type of images and to a greater extent in the categorization task than in the detection task. Such data suggest two distinct, although associated, types of residual dysfunction in ON: a magnocellular pathway alteration and a more general (magno and parvocellular) visual dysfunction that could implicate the cognitive levels of visual processing.

## 1. Introduction

Optic neuritis (ON) is an acute inflammatory disease of the optic nerve that chiefly affects young women [1, 2]. Visual loss is often unilateral and rapid, can be partial or complete, and may be associated with retrobulbar pain [3]. The degree of visual loss is apparently unrelated to age, gender, or abnormalities on fundus examination [1]. Furthermore, when the pathologic eye is stimulated, electrophysiological examination usually reveals visual evoked potentials (VEPs) with increased latency and reduced magnitude. Whilst optic neuritis has multiple causes, the most common etiology is multiple sclerosis (MS), a nervous system disease that affects the brain and the spinal cord; indeed, in 20% of MS patients, an episode of ON is the presenting sign of the disease. Although visual function improves spontaneously and quickly, corticosteroid treatment (methylprednisolone administered intravenously) is often recommended as it may accelerate visual acuity recovery [4]. Recovery usually begins 2 weeks after the acute phase and is complete within 1 to 3 months in most patients following their first episode of ON [4,5]. The degree of recovery is apparently unrelated to age, gender, ethnicity, concurrent presence of MS, or mor-

ISSN 0953-4180/13/$27.50 © 2013 – IOS Press and the authors. All rights reserved phological abnormalities indicated by brain Magnetic Resonance Imaging (MRI) [6]. It has been suggested that the visual acuity at 1 month post-onset of the ON episode predicts the ophthalmologic outcome (visual acuity, and/or visual field mean deviation, and/or contrast sensitivity) at 6 months [7].

However, 6 months after the acute phase of ON, 56% of patients still complain about the quality of their vision (according to their responses to a questionnaire). Among the patients that complain, 20% do not exhibit any observable ophthalmologic abnormality in terms of visual acuity, contrast sensitivity, visual field mean deviation, or color vision [8]. Similarly, the visual complaint is greater than expected (based on visual acuity) in patients with MS (with or without previous optic neuritis episode) [9]. Several factors could contribute to visual dysfunction following ON, including physiological, structural and functional neuro-ophthalmological changes observed in patients with or without complete visual recovery. As revealed by optical coherence tomography (OCT), which assesses macular volume and retinal nerve fiber layer (RNFL) thickness, most patients suffer from axonal loss within 3 to 6 months after the acute phase [10,11]. Specifically, central fibers of the optic nerve seem to exhibit a greater sensitivity to demyelination [12] leading to optic nerve atrophy, as reported in MRI studies [13,14].

The fibers that emanate from the retina are organized into two functionally and anatomically distinct pathways: the magnocellular pathway, which preferentially conveys low spatial frequency (LSF) and high temporal frequency (this encompasses most motion related information); and the parvocellular pathway, which preferentially conveys high spatial frequency (HSF) and low temporal frequency[1] (see Fig. 1) [15,16]. Interestingly, most of the central fibers in the optic nerve transmit detailed information. Considering data from various psychophysical and behavioral studies, some researchers have postulated that ON implies a specific defect in the parvocellular pathway [17,18], whereas other researchers have proposed an alteration of the magnocellular fibers in ON [19,20]. However, neither of these hypotheses has been unequivocally proven. Indeed, there remains some debate as to whether magnocellular or parvocellular fibers are equally affected in ON, or whether one of the pathways is more susceptible to damage [21]. One method to solve this question is to experimentally exploit the type of information each pathway conveys: global information in LSF in the magnocellular pathway vs. detailed information in HSF in the parvocellular pathway. In healthy participants, LSF rapidly reach higher-order cortical areas (parietal and temporal cortices), whereas HSF are transmitted more slowly to these areas (for further details, see [16]). Regarding the nature of the information transmitted (i.e. global vs. local), this means that there is a coarse-to-fine time course in spatial frequency processing [22]. This type of approach has also been used in a study of ON patients to whom stimuli were presented in the central visual field and in which LSF processing was again reported to be abnormally slow [23].

In the present study, we presented the participants with filtered (LSF or HSF) and unfiltered images during a rapid visual detection task (Is there a picture on the screen?) and a rapid visual categorization task (Is the picture a city or a highway?). In this study, we aimed to understand to what extent both a subclinical spatial frequencies processing deficit and the cognitive load of the visual task could explain the patients' complaints after apparent visual recovery.

## 2. Materials and methods

### 2.1. Participants

The study participants included 16 healthy individuals (10 females, 6 males; 28.20 $\pm$ 5.28 years old) and 8 patients (4 females, 4 males; 32.98 $\pm$ 5.78 years old) recovering from a first episode of ON. Patients completed the task approximately 1 month after their acute phase of the ON episode (mean interval between the last intravenous injection of methylprednisolone [IV-MP] and the study: 34 $\pm$ 3 days). At that time, they showed normal values for the following clinical parameters: visual acuity (Monoyer chart) (0.99 $\pm$ 0.11 and 1.05 $\pm$ 0.09 respectively with their pathologic eye and healthy eye), visual field extent (Humphrey 24-2 SITA-FAST program) (-mean deviation: $-1.17 \pm 1.01$ and $-0.84 \pm 1.05$ respectively with their pathologic eye and healthy eye), contrast sensitivity [24] (1.80 $\pm$ 0.15 and 1.86 $\pm$ 0.15 respectively with their pathologic eye and healthy eye), and fast RNFL thickness (measured by optical coherence tomography [OCT] using a Zeiss Stratus OCT$^{TM}$ system) (98.53 $\pm$ 7.86 and 101.16 $\pm$ 10.21 respectively with their pathologic eye and healthy eye). All but one patient (who received

[1] Although such dichotomy is not absolute, the relative 'weight' of each pathway depending on the spatial-temporal characteristics of the stimulus.

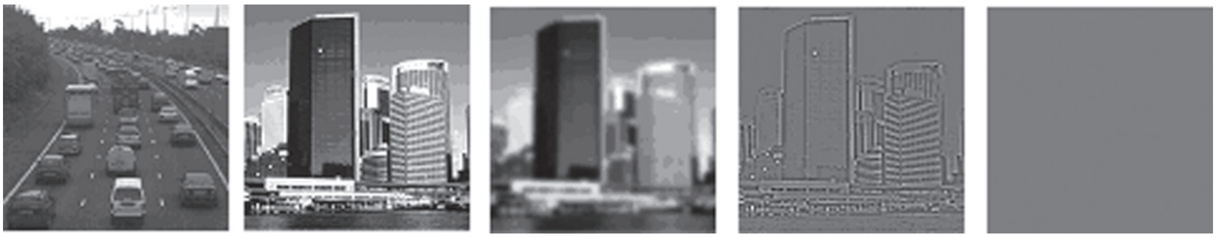

Fig. 1. Examples of stimuli used in the detection and categorization tasks. From left to right: two unfiltered images (highway and city); image filtered into low spatial frequency (< 4 cycles/degree) and high spatial frequency (> 6 cycles/degree); null stimulus.

5 g) were treated with 3 g of IV-MP, followed by oral prednisone for 2 weeks (progressively decreasing dosage: 60 mg/day for 5 days, 40 mg/day for 5 days and 20 mg/day for 5 days). Note that half of the patients subsequently developed MS. All participants were right-handed (as assessed with the questionnaire of Dellatolas et al. [25]) and had normal or corrected-to-normal visual acuity (decimal fraction: 0.99 with the pathologic eye, 1.05 with the healthy eye in patients). Patients showed a normal cognitive state (as measured with the Mini Mental State Examination; [26]; 29.25 $\pm$ 0.71) as did healthy controls (29.50 $\pm$ 0.97). All participants had high education levels (as determined with the French scale presented in Gil, 1996; [27]; 6.75 $\pm$ 0.71 for patients and 6.94 $\pm$ 0.25 for healthy controls). The two groups were similar in age ($t(22) = -2.03$; $p > 0.05$), sex ratio (chi$^2$, $p > 0.10$), cognitive state ($t(22) = 0.65$; $p > 0.05$), and education level ($t(22) = 0.96$; $p > 0.05$).

### 2.2. Material

The study design and the stimuli used have been described in detail in a previous study (on normal and brain-damaged patients) [28]. Briefly, eight black-and-white (256 grey-scale) natural scene images (six cities and two highways; see Fig. 1 for examples), from the Computational Visual Cognition Laboratory (available online at http://cvcl.mit.edu/database.htm, Oliva A., MIT; Cambridge, MA, USA), were selected and filtered (using Gaussian filters) to create LSF (spatial frequency content < 4 cycles/visual angle) and HSF (spatial frequency content > 6 cycles/visual angle) stimuli. Additionally, one grey image (256 grey-scale) was used as a null stimulus (see Fig. 1). All stimuli were presented surrounded with a black frame, such that their final size was 264 $\times$ 264 pixels. From these 25 different stimuli (8 images $\times$ 3 image types + 1 null stimulus), three blocks of ten stimuli (6 cities + 2 highways + 2 null stimuli) were created, each of which was specific to an image type (i.e. unfiltered, LSF or HSF images). Each block was repeated four times within a task to create 120 trials per task. Each trial began with a central fixation cross (600 ms), followed by the stimulus (100 ms), and finally, a grey answer-screen (1000 ms). The inter-trial interval was fixed at 900 ms.

### 2.3. Tasks and procedure

Participants sat facing the computer screen with their head fixed in a constant position using a chinrest. Stimuli were displayed centrally on a computer monitor (size: 19 in.; resolution: 1027 $\times$ 768 pixels), on a grey background, using E-Prime 1 software (E-prime Psychology Software Tools Inc., Pittsburgh, PA, USA). The screen was located 110 cm from the participant and the stimuli size was 4° of visual angle. Participants completed two tasks: *detection* and *categorization*. In the detection task, they were asked to press one button when an image was present on the screen and another button when the null stimulus was presented. In the categorization task they were required to press one button when they identified an image of a city and another one when they detected an image of a highway or the null stimulus. Participants used their forefinger and middle finger of their right (dominant) hand to respond by pressing a button on a SR-BOX aligned with their mid-sagittal plane. The task was completed twice in monocular vision (the unused eye was patched): once with the right eye and once with the left eye. The order of tasks, of blocks within a task and of eyes (right/left or healthy/pathologic), as well as the finger-to-stimuli association (i.e. image/city and forefinger vs. image/city and middle finger) were counterbalanced across participants within each group. Response quality (i.e. accuracy) and response times (RTs; in msec) were recorded.

### 2.4. Statistical analyses

The present study was designed to investigate the effect of stimulus type (low or high spatial frequencies) and task instruction (detection or categorization)

on performance and thus included null stimuli, highways, and cities images. However, in order to only test the effect of task instruction (as suggested in previous studies [29]) and avoid confounding effect of stimulus category (city or highway), only the performance related to cities was analyzed. In this way, images of null stimuli as well as highway images in the categorization task were considered as catch-trials. Response quality was ascertained based on the accuracy rate (i.e., the number of correct responses divided by the number of trials with an image). All statistical analyses were performed with the Statistica software package (release 7.1, 2006), with an alpha-level fixed at 0.05.

In a first step, data from the right and the left eye of healthy controls were compared to ensure they can be collapsed. In a second step, data from right and left optic neuritis patients were compared in respect to their pathologic or healthy eye here again to ensure data from the pathologic (or healthy eye) of both patients groups can be collapsed.

In healthy controls, for the accuracy measure, two separate analyses of variance (ANOVA) were run, because at least one dependant variable (task by image type) has no variance. The first ANOVA was run on mean accuracy per type of images (i.e., both tasks taken together), based on two groups (right and left eyes) and three image types (unfiltered, LSF or HSF images). The second ANOVA was run on the mean accuracy per task (i.e., all image types taken together), based on two groups (right and left eyes) and two tasks (detection and categorization). None of these two ANOVAs showed a significant main group effect or any significant interaction with the group factor (all $p > 0.05$). For the RTs measure, another ANOVA was run, based on two groups (right and left eyes), two tasks (detection and categorization), and three image types (LSF, HSF or unfiltered images). Again, no significant main group effect or significant interaction with the group factor (either two-level or three-level) was observed (all $p > 0.30$). Data from the right and left eyes were then collapsed to generate a *Control Eye* group, from which accuracy and RTs were measured.

Due to the small number of ON patients (four patients with their right eye affected and four patients with their left eye affected), non-parametric statistical analyses were used to assess differences between right and left pathologic eyes (of right and left ON patients, respectively), and then left and right healthy eyes (of right and left ON patients, respectively). Data from the pathologic eye of each patient, followed by data from their healthy eye, were analyzed using the Mann-Whitney U-test, whereby right ON patients were compared with left ON patients in each of the six conditions (2 tasks x 3 image types). No significant group effect was observed for either accuracy or RT measures (all $p > 0.30$). This led to creation, for accuracy and RT measures, of a *Pathologic Eye* group, based on data issued from the pathologic eye of patients, and a *Healthy Eye* group, based on data from the healthy eye of patients.

Mean accuracy and RTs from the control, pathologic, and healthy eyes were then further analyzed using an ANOVA with the two tasks (detection and categorization) and the three image types (unfiltered, LSF and HSF) as within-subject factor. When required, the specific effect of each factor was determined by *post-hoc* analysis (the Least Square Difference test).

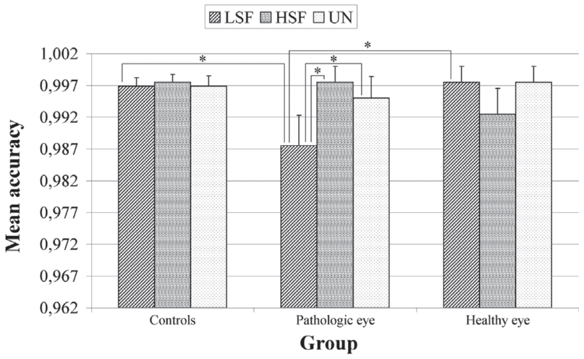

Fig. 2. Mean accuracy (ratio of correct responses) observed in each group (Control Eye [healthy individuals], Pathologic Eye [ON patients], and Healthy Eye [ON patients]) by image type (low spatial frequencies [LSF], high spatial frequencies [HSF] and unfiltered [UN]) for both tasks (detection and categorization) taken together. With their pathologic eye, the ON patients showed lower accuracy with LSF images than with UN images. Moreover, when LSF images were presented, the Pathologic Eye group had lower accuracy than did either the Healthy Eye group or the Control Eye group. *Significant difference ($p < 0.05$, post-hoc analysis).

## 3. Results

### 3.1. Accuracy

The only significant effect revealed by the ANOVA for three groups, two tasks and three image types was the group by image type interaction ($F(4,58) = 2.6$; $p = 0.046$). Post-hoc analyses (LSD-test) revealed a lower accuracy rate in the pathologic group with LSF images than in any other group or condition (all $p < 0.05$) (Fig. 2).

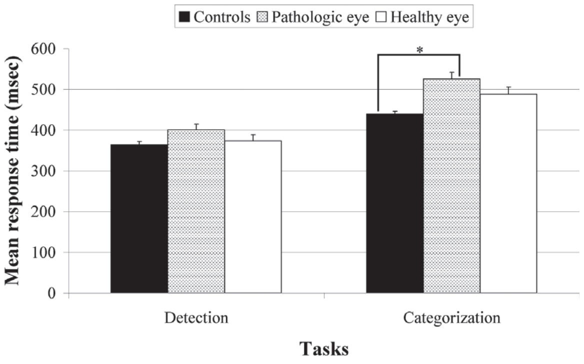

Fig. 3. Mean response times (RTs, msec) observed in each group (Control Eye [healthy controls], Healthy Eye [ON patients] and Pathologic Eye [ON patients]) in the detection task and in the categorization task, all image types taken together. In the categorization task, RTs were longer in the Pathologic Eye group than in the Control Eye group. *Significant difference ($p < 0.05$; *post-hoc* analysis).

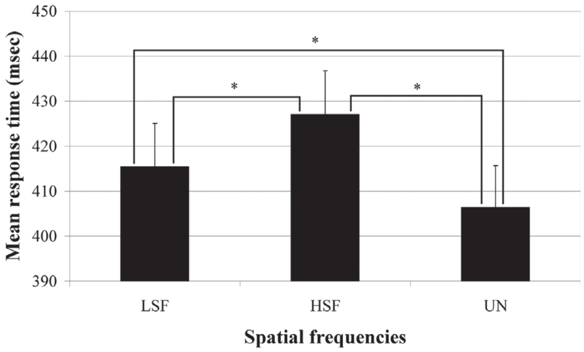

Fig. 4. Mean response times (RTs, in msec) observed for each image type (low spatial frequencies [LSF], high spatial frequencies [HSF] and unfiltered [UN] images) both tasks (detection and categorization task) and all groups taken together. Regardless of the task and of the group, RTs were longer with HSF images than with either LSF or UN images. Furthermore, RTs were longer for LSF images than for UN images. *Significant differences ($p < 0.05$; *post-hoc* analysis)

### 3.2. Response time

The ANOVA for three groups, two tasks and three image types showed a significant main task effect ($F_{(1,29)} = 151.32$; $p < 0.001$) modulated by a significant group by task interaction ($F_{(2,29)} = 3.84$; $p = 0.033$), as well as a main image-type effect ($F_{(2,58)} = 24.91$; $p < 0.001$).

*Post-hoc* analysis (LSD-test) indicated that within each group, RTs were longer in the categorization task than in the detection task ($p < 0.001$). Although the three groups did not differ in the detection task (all $p > 0.20$), the Pathologic Eye group showed longer RTs than did the Control Eye group in the categorization task ($p = 0.014$; note that the Healthy Eye group showed intermediate RTs, as it did not differ from either of the other two groups) (Fig. 3).

Regarding the effect of image type (Fig. 4), *post-hoc* analysis revealed that RTs were longer with HSF images than with LSF images, and were longer with either type of filtered image than with unfiltered images (all $p < 0.05$).

## 4. Discussion

In the present study, we sought to assess spatial frequency processing in ON patients in order to determine whether the magnocellular or parvocellular pathway is more altered after recovery from a first episode of the disease. In addition, we aimed to test to which extent the cognitive load of the task could affect performance. Overall, the present results suggest that LSF processing is preferentially affected, which is consistent with a dysfunction in the magnocellular pathway. As we will discuss below, the present findings suggest that this specific residual magnocellular deficit could be associated to a more general dysfunction related to the attentional and cognitive load of the visual task.

*In terms of accuracy*, ON patients showed a specific deficit when processing LSF images with their pathologic eye, but HSF processing was preserved. Although this result may be considered preliminary because of the small number of patients, our findings closely parallel those of Haupt et al. [23]. In their study and in ours, processing of spatial frequencies higher than 6 cycles per degree was preserved; however, processing of spatial frequencies lower than 6 cycles per degree (in their study), or even lower than 4 cycles per degree (in our work) was altered.

The present data are also consistent with previous findings showing impaired motion [19,30,31] or achromatic stimuli [20] processing, thus also suggesting a magnocellular deficit among ON patients that appeared to have full ophthalmologic recovery. Magnocellular deficits have been proposed to represent a behavioural correlate of demyelination in ON patients [31,32]. However, it is interesting to note that in ON patients, the deficit in processing LSF only occurs in terms of error rate and not in terms of reaction time. In this way, if there is a magnocellular deficit in ON patients, this deficit is not responsible for a specific increase of the response speed when processing LSF in a visual detection task. The only significant RT difference between control and ON participants was observed in the categorization task. Indeed, in the categorization task, RTs were longer in ON participants with their affected eye than in controls regardless of the spatial frequency content of images.The present results thus point to a more cognitive visual deficit independent of spatial frequency content when the patient has to rapidly use visual information in a complex visual task. Such data is consistent with the assumption of two distinct yet associated types of dysfunction in ON: a magnocellular pathway alteration and a more general (magno and parvocellular) visual dysfunction that could imply the attentional and semantic aspects of visual cognition. However, further research is needed to disentangle several hypotheses. Indeed, on one hand, knowing that some of these patients might subsequently develop a MS disease, the present cognitive visual deficit could be seen as the consequence of an early cortical damage. On the other hand, the present findings could reflect a cortical reorganization as several authors suggested [6,33–36]. Altogether, further behavioural and neuroimaging research is required to better ascertain the contribution of peripheral and cortical visual pathways modifications in ON patients with or without complete visual recovery and to investigate to what extent cortical reorganization is necessary for recovery or reflects a maladaptive process. Regarding the clinical management of ON patients, the role of corticosteroid therapy alone in reducing the risk of subsequent MS is unclear, but recent studies suggest that the combination of immunomodulation agents (IMAs) and corticosteroids significantly reduces the later development of MS [37]. In addition, a review of different studies by Johnson and Morey also underscores the need to investigate the efficacy of high-dose corticosteroid to reduce the occurence of MS in ON patients [38].

## Acknowledgements

Publication of this article was supported by the Caesarea Edmond de Rothschild Foundation (Israel) and the Memorial A. De Rothschild (Geneva, Switzerland).

The authors indicate no financial conflict of interest.

Involved in Design of study (S.C., O.C.); Conduct of study (S.C.); Data collection (A-C.V.); Data management (A-C.V., C.C., O.C.); Data analysis (A-C.V., C.C., O.C.); Data interpretation (A-C.V., C.C., O.C., S.C.); Preparation of manuscript (A-C.V., C.C., S.C.); Review of manuscript (A-C.V., C.C., O.C., S.C.); and Approval of manuscript (A-C.V., C.C., O.C., V.V., C.V., O.G., S.C.).

All procedures were conformed to the Declaration of Helsinki. Informed consent was obtained from the each of the patients participating in the study.

The authors thank E. Marcellesi for assessing some of the healthy controls.
